# Dengue viruses infect human megakaryocytes, with probable clinical consequences

**DOI:** 10.1371/journal.pntd.0007837

**Published:** 2019-11-25

**Authors:** Megan B. Vogt, Anismrita Lahon, Ravi P. Arya, Jennifer L. Spencer Clinton, Rebecca Rico-Hesse

**Affiliations:** 1 Integrative Molecular and Biomedical Sciences Graduate Program, Baylor College of Medicine, Houston, Texas, United States of America; 2 Department of Molecular Virology and Microbiology, Baylor College of Medicine, Houston, Texas, United States of America; Duke-NUS GMS, SINGAPORE

## Abstract

One of the most important clinical signs of dengue virus infection is the reduction of white blood cells and platelets in human peripheral blood (leukopenia and thrombocytopenia, respectively), which may significantly impair the clearance of dengue virus by the immune system. The cause of thrombocytopenia and leukopenia during dengue infection is still unknown, but may be related to severe suppression of bone marrow populations including hematopoietic stem cells and megakaryocytes, the progenitors of white blood cells and platelets respectively. Here, we explored the possibility that bone marrow suppression, including ablation of megakaryocyte populations, is caused by dengue virus infection of megakaryocytes. We used three different models to measure dengue virus infection and replication: *in vitro*, in a human megakaryocyte cell line with viral receptors, *ex vivo*, in primary human megakaryocytes, and *in vivo*, in humanized mice. All three systems support dengue virus infection and replication, including virus strains from serotypes 1, 2, and 3, and clinical signs, in vivo; all assays showed viral RNA and/or infectious viruses 7–14 days post-infection. Although we saw no significant decrease in cell viability *in vitro*, there was significant depletion of mature megakaryocytes *in vivo*. We conclude that megakaryocytes can produce dengue viruses in the bone marrow niche, and a reduction of cell numbers may affect bone marrow homeostasis.

## Author summary

Dengue virus is the most common cause of viral hemorrhagic fever in humans. Over half of the world’s population lives in an at risk area for dengue virus infection, and this number will continue to grow as climate change allows the mosquito vectors of dengue virus to expand their breeding ranges to more temperate climates. Currently, there are no specific treatments for dengue virus infection. Understanding how dengue virus causes hemorrhagic fever could inform the development of these much needed treatments. Populations of important immune system mediators, such as white blood cells and platelets, are significantly dysregulated during dengue virus infection. These cells originate in the bone marrow, which experiences significant suppression, including a complete ablation of megakaryocytes (platelet progenitor cells), during DENV infection. Here, we add to the knowledge on how dengue virus induces bone marrow suppression by investigating whether dengue virus infects human megakaryocytes. We discovered that dengue virus infects human megakaryocytes *in vitro*, *ex vivo*, and *in vivo* models of dengue virus infection; however, dengue virus infection does not appear to directly affect viability of human megakaryocytes. Future studies will investigate whether infected megakaryocytes are still able to perform their functions of producing platelets and maintaining bone marrow homeostasis.

## Introduction

Dengue virus (DENV; *Flavivirus*) is the most common cause of hemorrhagic fever in humans, worldwide. Each year, approximately 390 million DENV infections occur [1], with an estimated 50 million becoming symptomatic, 500,000 progressing to hemorrhagic fever, and 50,000 resulting in death [2, 3]. In addition, DENV causes a significant economic burden, with an estimated $9 billion (USD) spent each year treating DENV infections [4]. Furthermore, the World Health Organization (WHO) estimates that over half of the world’s population (4.5 billion people) live in an area at-risk for DENV infection, including residents of the southern United States and US territories [5]. This number is expected to increase as climate change allows for the expansion of the host range of DENV’s primary vector, the *Aedes aegypti* mosquito [2]. There are currently no DENV vaccines approved for all persons, and no specific anti-DENV treatments [6, 7]. Understanding the mechanisms leading to DENV disease will allow for the production of more effective DENV vaccines and treatments.

The onset of DENV symptoms occur 5 to 8 days following an infected mosquito bite [8]. Most symptomatic DENV infections result in a self-limiting febrile illness that lasts 3 to 7 days and is characterized by maculopapular rash, retro-orbital pain, arthralgia, and myalgia. Approximately 1% of symptomatic DENV infections will progress to hemorrhagic fever upon defervescence and clearance of DENV from the blood [8]. Dengue hemorrhagic fever is a potentially life-threatening condition characterized by excessive bruising, plasma leakage, organ hemorrhaging, bloody vomit and stool, and hypovolemic shock. These hemorrhagic manifestations are likely not caused by severe damage to the endothelium, because endothelial damage has not been observed upon autopsy of humans who succumbed to DENV infection [8].

Platelets are crucial in maintaining vascular homeostasis and preventing spontaneous bleeding in otherwise healthy individuals [9]. A significant reduction in platelet counts (thrombocytopenia) often occurs during DENV infection and ranges from mild (50,000–150,000 platelets/μL blood) in cases of dengue fever to severe (<50,000 platelets/μL blood) in cases of dengue hemorrhagic fever [5, 6]. Peak thrombocytopenia occurs simultaneously with defervescence and the onset of dengue hemorrhagic fever [8, 10]. Thus, severe thrombocytopenia in DENV infections may play a crucial role in the development of hemorrhagic manifestations. However, platelet transfusions are contraindicated for treatment of dengue hemorrhagic fever and may increase severity of disease [5, 11]. Platelet functions are dysregulated during DENV infection, including increased platelet activation, clot formation, apoptosis, and inflammatory cytokine production, all of which contribute to thrombocytopenia [12–17]. Instead of contributing to hemorrhagic manifestations, thrombocytopenia during DENV infection may indicate widespread hematological dysregulation.

Platelets are not the only hematopoietic population dysregulated during DENV infection. Leukopenia (decreased white blood cell counts) and lymphocytosis (increased lymphocyte counts), especially of atypical B and T cell populations, also occur during DENV infection and are particularly severe in dengue hemorrhagic fever [8, 18]. Like peak thrombocytopenia, peak leukopenia and lymphocytosis occur coincident with defervescence and onset of dengue hemorrhagic fever [8]. The development of hematopoietic cells occurs in the bone marrow. Bone marrow suppression occurs in DENV infection and peaks approximately 2–3 days before peak thrombocytopenia and leukopenia [10]. This suppression affects all cell populations in the bone marrow including a complete ablation of mature megakaryocytes, platelet progenitor cells [10]. While the primary role of megakaryocytes is to produce platelets, recent studies indicate that megakaryocytes are integral to maintaining the homeostasis of various processes within the bone marrow including hematopoiesis, plasma cell (terminally differentiated, antibody secreting B cells) maintenance, and skeletal homeostasis [19–23]. Thus, megakaryocyte suppression during DENV infection may impact counts of platelets and other hematopoietic cells, potentially impacting disease severity. Understanding how DENV infection causes megakaryocyte suppression may allow for the creation of preventive or treatment measures for DENV.

Previous studies investigating bone marrow and megakaryocyte suppression during DENV infection have found that DENV reduces the number of human megakaryocytes and their progenitor cells in infected humanized mice [24], megakaryocytes from non-human primates are susceptible to DENV infection *in vivo* and *ex vivo* [25], and DENV infects bone marrow cells in both humanized mice and non-human primate models [24, 26]. Infection of human megakaryocytes *in vivo* has not yet been confirmed, although CD61+ cells (megakaryocytes) isolated from healthy human bone marrow are susceptible for DENV infection *ex vivo* [25]. An additional study discovered large, DENV+, CD61+ cells in peripheral blood in humans infected with DENV [27]; however, a literature review found no other instances of human megakaryocytes found in peripheral blood. These CD61+ cells are likely infected platelets adhered to leukocytes [28].

We hypothesized that DENV causes megakaryocyte suppression, in part, through direct infection of megakaryocytes; therefore, the goal of this study was to measure DENV infection of megakaryocytes in different systems. We used *in vitro* (UT-7 cells), *ex vivo* (megakaryocytes differentiated from human CD34^+^ cells) and *in vivo* (humanized NOD/SCID/IL-2γ^-/-^ (hu-NSG) mice) models of human megakaryocytes and DENV infection. The results of this study provide insights into the mechanisms behind bone marrow and megakaryocyte suppression during DENV infection and sets the stage for future studies of whether DENV directly impairs hematopoietic cell populations.

## Materials and methods

### Dengue virus strains

Dengue serotype 1 (DENV-1) strain VN/BID-V1792 was obtained from BEI (NR44083) at C6/36 passage 1, and we passaged twice in C6/36 cells. Dengue serotype 2 (DENV-2) strain K0049 was isolated from serum of a DENV patient in Thailand in 1995, and has been used extensively in our laboratory (BEI NR12215). It has been passaged three times in C6/36 cells. Dengue serotype 2 strain 16681 is a laboratory-adapted strain of DENV and was obtained from Richard Kinney (CDC); it has been passaged through monkey kidney cells >10 times, live rhesus macaque p.1, whole mosquito p.2, and C6/36 cells p.5. Dengue serotype 3 (DENV-3) strain 7431/98 is a clinical isolate from a DENV outbreak in Nicaragua in 1998, obtained from Dr. Eva Harris (UC Berkeley). It was passaged twice in C6/36 cells. All virus strains were propagated in C6/36 cells and frozen in 30% gelatin.

UV-inactivated K0049 was used as a negative control in all cell culture experiments. Five hundred microliters of K0049 was transferred to each well of a 6-well plate. The plate was then positioned in the center of UV-Stratalinker 1800 (Stratagene) and the lid removed. Using the energy setting, 0.9J UV was applied to the virus four times, for a total of 3.6J UV exposure. Fresh UV-inactivated virus was prepared for each infection. Following preparation, UV-inactivation was confirmed by endpoint dilution assay (described below).

### Humanized-NSG mice

Humanized NOD/SCID/IL-2γ^-/-^ (hu-NSG) mice were prepared as described previously [29]. Briefly, male and female NSG mice were obtained from Jackson Labs (Bar Harbor, ME) and were bred at Baylor College of Medicine, in the Transgenic Mouse Facility (TMF). When pups were 1 day old, they were sublethally irradiated with 100 centigrays from a cesium source at TMF, and injected intrahepatically with 300,000 human CD34^+^ stem cells obtained from human umbilical cord vein blood (University of Texas MD Anderson Cord Blood Bank).

Eight weeks following injection, mice were checked for human immune cell engraftment via flow cytometry. Approximately 100μL of blood were obtained via retroorbital bleed. Antibodies against human CD45 (clone HI30; APC; BD Biosciences) and mouse CD45 (30F11; FITC; Miltenyi Biotec) were added to blood samples and incubated 30 minutes. Red blood cells were lysed using BD FACS Lysing Solution (BD Biosciences) according to the manufacturer’s instructions. Samples were run on an LSRII flow cytometer (BD) and analyzed using FlowJo software (v10, TreeStar). Human engraftment percentage was determined by dividing the number of human CD45^+^ cells by the sum of human and mouse CD45^+^ cells. Only mice with a human engraftment percentage of at least 20% were used in this study.

### Dengue infection of humanized mice

Hu-NSG mice were infected with 10^6^ plaque forming units of virus delivered via subcutaneous injection. Clinical signs of infection were assessed on days 2, 4, 6, 8, 10, 12, and 14 post infection. Mice were anesthetized via isoflurane inhalation, and temperature and erythema were measured via rectal thermometer and DSMII Colormeter (Cortex Technologies), respectively. Approximately 20μL of blood were obtained via retroorbital bleed to quantify viremia. Blood was transferred to microcentrifuge tubes and allowed to clot before centrifuging at 500g for 15 minutes to separate out serum. Trizol LS (Ambion) was added to serum samples according to manufacturer’s instructions, and samples were stored at -70°C until RNA extractions and qRT-PCR were performed. On day 10 post infection, blood was collected via retroorbital bleed to perform platelet counts.

On days 8 and 14 post infection, mice were euthanized via isoflurane overdose. Femurs were dissected out of euthanized mice, and bone marrow was flushed out of femurs using a 5/8 inch 25 gauge needle and sterile PBS with 2% FBS. Marrow was forced through a 0.45μM filter to create a single cell suspension. Cells were treated with red blood cell lysis solution (eBioscience) and washed with sterile PBS/FBS. Infected megakaryocytes were identified using flow cytometry.

### Isolation and differentiation of human CD34^+^ cells

Human umbilical cord vein blood was obtained from the MD Anderson Cancer Center Cord Blood Bank. Blood was diluted 1:2 in sterile PBS and separated using Leucosep tubes (Grenier Bio-One) and Lymphocyte Separation Media (Corning) according to the manufactures’ instructions. Following separation, the lymphocyte layer was treated with red blood cell lysis solution (eBioscience) and washed in PBS/FBS. CD34^+^ stem cells were isolated using the EasySep Human CD34 Positive Selection Kit (Stem Cell Technologies) according to the manufacturer’s instructions.

Isolated CD34^+^ cells were plated in 24 well plates at 40,000 cells/mL in IMDM media (Gibco) containing 20% BIT Serum substitute (Stem Cell Technologies), 20μg/mL Human LDL (Stem Cell Technologies), 100μM 5x10^-2^M β-Mercaptoethanol (Invitrogen), and 1× StemSpan Megakaryocyte Expansion Supplement. Cells were incubated at 37°C at 5% CO_2_ in a humidified incubator. Four days post isolation, cells were split 1:2. Seven days post isolation, cells were replated at 3 to 5x10^5^ cells/ml. Differentiation was assessed on days 4, 7, 11, and 14 post isolation using flow cytometry [30].

### Growth and maintenance of cell lines

UT-7 cells (human megakaryocytic cell line) were obtained from DSMZ-German Collection of Microorganisms and Cell Cultures (Braunschweig, Germany; Catalog number: ACC-137). Cells were grown according to manufacturer’s instructions. Briefly, following revival from liquid nitrogen, cells were cultured in MEMα–no nucleosides (Gibco) containing 40% FBS and 5ng/mL GM-CSF (Peprotech) for 6 days, after which FBS was reduced to 20%. Cells were split every three days, and replated at 5x10^5^ cells/mL in fresh media. Cells were incubated at 37°C at 5% CO_2_ in a humidified incubator. All UT-7 cells used for experiments were below passage 25.

C6/36 cells (*Aedes albopictus* cell line) were obtained from ATCC (CRL-1600). Cells were cultured in Minimum Essential Media (HyClone) containing 10% FBS, 1% Sodium Pyruvate (Sigma), 1% MEM Non-Essential Amino Acids (Sigma), and 1% Penicillin/Streptomycin/L-Glutamine solution (Sigma). Cells were incubated at 29°C at 5% CO_2_ in a humidified incubator.

### Infection of human megakaryocytes and UT-7 cells

Human megakaryocytes were infected at day 7 post isolation; at this time point the cell population was comprised mostly of immature megakaryocytes, based on flow cytometry for cell surface marker expression. UT-7 cells were infected at least three days after FBS in the media was reduced from 40% to 20%. Both cell types were infected in a similar method. Cells were transferred to 96 well plates with 2x10^5^ cells/well. Cells were centrifuged at 200g for 5 minutes, and cell culture media was discarded. Cells were resuspended in 100μL of DENV diluted to the appropriate multiplicity of infection (MOI) in cell culture media containing 2% FBS or BIT Serum Substitute. Cells were incubated at 37°C at 5% CO_2_ in a humidified incubator for 2 hours, rocking every 15 minutes. Following incubation, cells were washed three times in sterile PBS and resuspended in 200μL of appropriate cell culture media containing 2% FBS or BIT Serum Substitute. UT-7 cells were transferred to 48 well plates. Human megakaryocytes were transferred to 24 well plates and media was added to each well for a final volume of 500μL media per well. Both cell types were incubated at 37°C at 5% CO_2_ in a humidified incubator.

Daily, following infection, 10% of total media volume was removed from both cell types, treated with Trizol LS (Ambion), and stored at -70°C for later quantification of viral RNA via qRT-PCR. Fresh media was added in a volume equaling the amount removed. Supernatants from UT-7 cells were collected daily for later assessment of infectious virus. The entire well volume was transferred to a microcentrifuge tube, and cells were pelleted by centrifuging at 200g for 5 minutes. Supernatants were transferred to a new tube, and 30% gelatin was added. Samples were stored at -70°C until presence of infectious virus could be determined via end point dilutions on C6/36 cells. The remaining cell pellet was used to assess cell viability and percentage of infected cells via flow cytometry.

### Quantification of viral RNA

RNA was extracted from serum samples from hu-NSG mice and cell supernatants from human megakaryocyte and UT-7 cells according to Trizol LS (Invitrogen) manufacturer instructions. RNA pellets were resuspended in 50μL DEPC treated water (Ambion). Viral RNA was quantified via a one-step qRT-PCR [31] using TaqMan fast virus one step master mix (Applied Biosystems) per the manufacturer’s instructions. Ten microliters of RNA were used per reaction. Table 1 lists the primer and probe sequences and concentrations. Cycling conditions are described in Table 2. The amplification products are approximately 100bp regions of the capsid protein that are unique to each serotype. An absolute standard curve of *in vitro* transcribed RNA standards from DENV-2 was used to determine concentration of viral RNA in samples. This assay can detect a minimum concentration of 240 RNA copies per mL or 1.2 μL per reaction.

**Table 1:** Primer and probe sequences used in this study.

Serotype	Forward Primer	Reverse Primer	Probe
DENV-1	ACC GTC TTT CAA TAT GCT GAA ACG	GGT ATG GCT AGA AAT CTT AGG AAT G	6FAM–TTC ACA GTT GGC GAA GAG ATT CTC AAA AGG AT–TAMRA
DENV-2	GCT GAA ACG CGA GAG AAA CC	CAG TTT TAT TGG TCC CG TCC CT	6FAM–AGC ATT CCA AGT GAG AAT CTC TTT GTC AGC TGT—TAMRA
DENV-3	GCT GAA ACG CGT GAG AAA CC	CAA TTT CAT TGG TCC CTG GCC GT	6FAM–AGC AGT CCT TTT GAG AAT CTC TTC–TAMRA

All sequences are listed 5’ to 3’. DENV-2 primer and probe sequences were obtained from [31]. All other primers and probes were designed using similar to logic that used in deriving primers for DENV-2.

**Table 2:** RT-PCR cycling conditions.

Step	Sub-step	Temperature (°C)	Time (s)	Repeated
Reverse Transcription	-	50	300	1x
Reverse Transcriptase Inactivation/Initial Denaturation	-	95	20	1x
Amplification	Denaturation	95	3	40x
	Annealing and Extension	60	30	

RT-PCR assays were run on a Step One Plus thermocycler (Applied Biosystems) using the fast plates and fast setting.

### Flow cytometry assays

#### Counting platelets in hu-NSG mice

On day 10 post infection, 10μL of blood were collected in heparinized tubes from each mouse via retroorbital bleed for counting platelets. Blood was transferred to 5mL round bottom tubes (BD Falcon) containing 600μL megakaryocyte buffer (0.1mM theophylline (Sigma), 15mM sodium citrate (Sigma), 1% BSA (Sigma) in sterile PBS) [30]. Antibodies against human CD41 (clone HIP8; PE; Biolegend) and mouse CD41 (clone MWReg30, BV510, Biolegend) were added, and samples were incubated 25 minutes at room temperature in the dark. Twenty-five microliters of CountBright absolute counting beads (Invitrogen) were added to each sample, and samples were incubated 60 minutes at room temperature in the dark. Samples were run on a LSRII (BD) flow cytometer, making sure to capture at least 1000 counting bead events per sample. Data were analyzed using FlowJo (TreeStar).

#### Identification of infected megakaryocytes in hu-NSG mice

Bone marrow cells isolated from hu-NSG mice were blocked with normal mouse serum (Invitrogen) for 10 minutes at room temperature. Samples were incubated for 30 minutes at 4°C with antibodies against human CD45 (clone 2D1, Amcyan, BD Biosciences), human CD41a (clone HIP8, PE, Biolegend), and human CD42b (clone HIP1, PerCP, Biolegend) diluted in megakaryocyte buffer. Then, samples were fixed and permeabilized using the Cytofix/Cytoperm Kit (BD Biosciences) according to the manufacturer’s instructions. Samples were incubated for 30 minutes at 4°C with antibody against DENV envelope protein (clone 4G2, FITC, Millipore). Samples were run on an LSRII (BD) and data were analyzed using FlowJo (TreeStar).

#### Determination of megakaryocyte differentiation status

Cells differentiated from human CD34^+^ cells were blocked with normal mouse serum (Invitrogen) for 10 minutes at room temperature. Samples were incubated for 30 minutes at 4°C with antibodies against human CD34 (clone 581, AF700, Biolegend), human CD41a (clone HIP8, PE, Biolegend), human CD42b (clone HIP1, FITC, Biolegend), and human CD62L (clone AK4, AF647, Biolegend) diluted in megakaryocyte buffer. Samples were run on an LSRII (BD) cytometer and data were analyzed using FlowJo (TreeStar). Cells that were CD41a^+^ CD42b^-^ were considered immature megakaryocytes and cells that were CD41a^+/-^ CD42b^+^ were considered mature megakaryocytes.

#### Determination of putative DENV receptors on UT-7 cells

UT-7 cells were blocked with normal mouse serum (Invitrogen) for 10 minutes at room temperature. Samples were singly incubated for 30 minutes at 4°C with antibody against human CD206 (clone 19.2, PE, BD Biosciences), human DC-SIGN (clone 120507, AF647, R&D Systems), human DC-SIGNR (clone 120604, AF594, R&D Systems), human Axl (clone MM0098-2N33, AF488, Novus Biologicals), human CD300a (MEM260, AF647, BD Biosciences), human TIM1 (clone 219211, AF700, Novus Biologicals), or Tyro3 (polyclonal, AF405, Novus Biologicals) diluted in PBS/FBS. Samples were run on an LSRII (BD) cytometer and data were analyzed using FlowJo (TreeStar).

#### Determination of UT-7 infection status and viability

UT-7 cells were stained with Ghostdye UV450 (Tonbo) per the manufacturer’s instructions. Cells were blocked with normal mouse serum (Invitrogen) for 10 minutes at room temperature. Then, cells were fixed and permeabilized using the Cytofix/Cytoperm kit (BD Biosciences) according to the manufacturer’s instructions. Samples were then incubated for 30 minutes at 4°C with antibody against DENV envelope (4G2, FITC, Millipore). Samples were run on an LSRII cytometer (BD) and data were analyzed using FlowJo (TreeStar). Viability was also assessed via trypan blue exclusion. A sample of infected UT-7 cells were collected every other day post infection and diluted in cell culture media with trypan blue (final dilution 1:10). Total cells, trypan blue stained cells, and unstained cells were counted via hemocytometer. Viable cells were those that were unstained by trypan blue. The percentage of viable cells was calculated by dividing the number of unstained cells by the number of total cells.

Gating strategies for all flow cytometry assays are described in S1 Text. All data are available at the flow cytometry repository: https://flowrepository.org/id/FR-FCM-Z2B4

### Immunofluorescence assay for detection of viral proteins in infected UT-7 cells

Labtek II CC2 coated chamber slides (Nunc) were treated with poly-D-lysine (300μL each well) and incubated 30 minutes at room temperature. Treated slides were left to dry overnight in a sterile environment. UT-7 cells were infected as described above. One day post infection, UT-7 cells were transferred to treated chamber slides. To assist in cell adherence, chamber slides were centrifuged at 200g for 5 minutes then incubated for 2 hours at 37°C with 5% CO_2_ in a humidified incubator. Chamber slides were centrifuged again and media was gently removed via pipette. Cells were fixed with 200ul per well of Cytofix/Cytoperm solution (BD Biosciences) for 30 minutes at 4°C. Next, cells were blocked in TBS Superblock (ThermoFisher) diluted 1:2 in TBS for 30 minutes at room temperature. Then, cells were incubated overnight at 4°C in antibody against DENV envelope (4G2, mouse IgG, Novus Biologicals) diluted 1:50 in Perm/Wash buffer (BD Biosciences).

The next day, cells were washed twice in PBS (BD Biosciences) and incubated for 2 hours at 4°C in the dark in anti-mouse IgG (AF488, ThermoFisher). Cells were washed twice and then incubated 10 minutes at room temperature in the dark with NucBlue ReadyProbes Reagent (Invitrogen) diluted 1:10 in PBS. Cells were washed once more. Chambers were removed from the chamber slide, and the slide dried for 10 minutes at room temperature to remove excess liquid. Approximately 15μl of Prolong Anti-Fade Mounting Media (Cell Signaling Technologies) were added per well. Bubbles were gently removed using a pipette tip. A glass coverslip was laid over the slide, and any air bubbles were gently pushed out. The slide was left to cure overnight at room temperature before sealing with clear nail polish. Images were taken using a Nikon A1SR microscope with NIS Elements software.

### Endpoint dilutions to measure infectious virus in cell supernatants

C6/36 cells were seeded at 1x10^5^ cells per well in a 48 well plate and incubated overnight at 29°C, 5% CO_2_ in a humidified incubator. The next day, UT-7 cell supernatants were thawed and ten-fold serially diluted in C6/36 growth media containing 2% FBS. C6/36 cells were infected by removing growth media and treating with 100μL of appropriate supernatant dilution per well. Cells were incubated for 2 hours at 29°C, 5% CO_2_ in a humidified incubator. Then, supernatants were pipetted off, and fresh C6/36 media (2% FBS) was added to each well. Cells were reincubated for 7 days.

Infected C6/36 cells were visualized via immunofluorescence against DENV envelope protein. Cell media were removed from wells, and cells were fixed and permeabilized using a 1:1 mix of ice-cold acetone (Fisher Scientific) and methanol (Fisher Scientific). Cells were incubated 20 minutes at -20°C before aspirating off acetone/methanol and drying for 20 minutes at room temperature in a chemical fume hood. Cells were blocked for 20 minutes at 37°C with TBS Superblock (ThermoFisher) diluted 1:2 in TBS. Then, cells were incubated overnight at 4°C with antibody against DENV envelope (4G2, mouse IgG, Novus Biologicals) diluted 1:50 in blocking buffer. The following day, cells were washed twice in TBS and incubated 2 hours at 37°C with antibody against mouse IgG (AF488, ThermoFisher). Then, cells were washed twice in TBS and incubated 10 minutes at room temperature in NucBlue Live ReadyProbes Reagent (Invitrogen) diluted 1:10 in TBS. Cells were washed again in TBS and left to dry overnight at room temperature. Results were visualized on a fluorescent microscope (Zeiss). A well was considered positive for infectious virus if there were any cells that had stained with 4G2. The endpoint dilution was noted as the highest dilution of supernatant that still yielded infected cells.

### Statistical analysis

Statistical analysis was performed using GraphPad Prism v8.0. Most data in this study were analyzed via two-way analysis of variance (ANOVA) followed by multiple comparisons t-test using the Holm-Sidak correction. Infectious virus immunofluorescent results were analyzed by hand using the Mann-Whitney rank-sum test as described in [32].

### Ethics statement

All experiments involving mice were done in accordance with guidelines of the Institutional Animal Care and Use Committee at Baylor College of Medicine (IACUC Protocol AN-6151), and the recommendations in the *Guide for the Care and Use of Laboratory Animals* (Institute for Laboratory Animal Research, National Research Council, National Academy of Sciences, 2011). The Institutional Review Board at Baylor College of Medicine determined that our use of human blood products in this study did not constitute human subjects research.

## Results

### Dengue virus infects and replicates in an *in vitro* model of human megakaryocytes

We began our investigation into whether DENV infects human megakaryocytes by using the UT-7 cell line as an *in vitro* model of human megakaryocytes. UT-7 cells are a human megakaryocytic cell line derived from bone marrow cells of a male who was suffering from acute megakaryoblastic leukemia [33]. UT-7 cells are similar to immature megakaryocytes in that they are polyploid and express the cell surface marker CD41a UT-7 cells may express CD42b, although expression is variable and dependent on spontaneous maturation of UT-7 cells[33]. We preferred the UT-7 cell model to other human megakaryocyte-like cell lines, Meg-01 and K562, because the latter cell lines are more closely related to common myeloid progenitor cells. Meg-01 and K562 cells do not express classical megakaryocyte cell surface markers (CD41a or CD42b) and can only be stimulated to be more like immature megakaryocytes through treatment with phorbol myristrate acetate (PMA) [34]. In addition, we focused on using DENV-2 strains, because this serotype was associated with the emergence of dengue hemorrhagic fever in the western hemisphere and has a long history of associated severe disease outbreaks [35–37].

**Fig 1:**
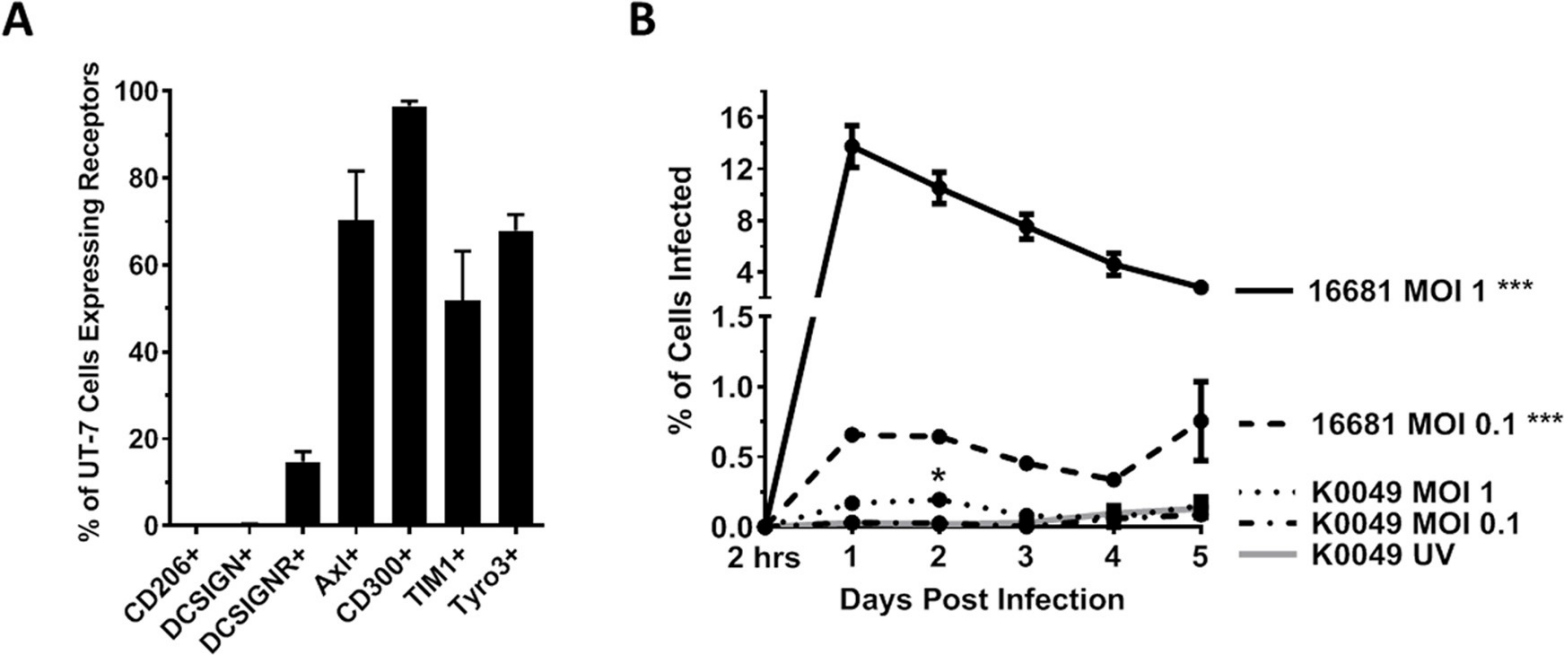
Dengue virus infects UT-7 Cells. (A) Using flow cytometry, UT-7 expression of putative DENV receptors were interrogated. Data from three independent experiments are represented as the mean percentage of the UT-7 cell population that was positive for a given receptor. Error bars are 1 standard error of the mean (SEM). (B) UT-7 cells infected with DENV-2 strains K0049 and 16681 were fixed, permeabilized, and incubated with antibody against DENV envelope protein. Samples were interrogated via flow cytometry, and data from three independent experiments are represented as the percentage of the UT-7 cell population that was infected (positive for DENV envelope). Error bars are 1 SEM. Statistical significance was determined using a two-way ANOVA and multiple comparisons t-test (with Holm-Sidak correction). Statistical significance next to the virus strain name indicate that that infection was significantly different from the UV-inactivated control as assessed by two-way ANOVA. Significance at a specific timepoint indicates that the condition at that timepoint was significantly different from the UV-inactivated control via t-test but that the overall infection was not significantly different via ANOVA. * p<0.05; **p<0.01; *** p<0.005; ****p<0.001.

**Fig 2:**
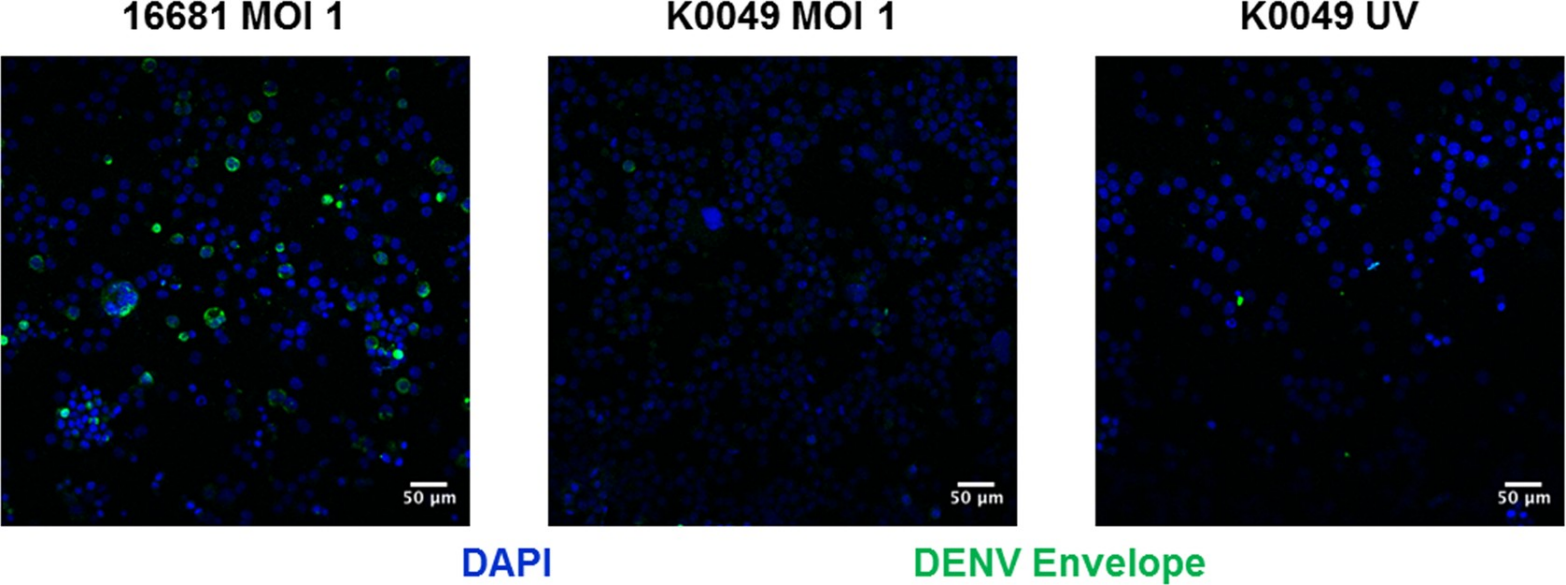
Dengue virus envelope protein is visualized in infected UT-7 cells. Representative immunofluorescence images of UT-7 cells infected with 16681 MOI 1, K0049 MOI 1, or UV-inactivated K0049 at day one post infection. Blue represents nuclei stained with DAPI. Green represents DENV envelope protein stained with the 4G2 monoclonal antibody. Scale bars are 50μM. Images were obtained using a Nikon A1SR microscope using NIS elements software.

We first wanted to determine whether DENV can enter UT-7 cells. The specific receptor that DENV uses to enter cells has yet to be determined; however, putative DENV receptors include C-type lectins (DC-SIGN, DC-SIGNR, and CD206) and phosphatidyl serine receptors (Axl, TIM-1, Tyro3, CD300a) [38, 39]. We used flow cytometry to determine that UT-7 cells express CD300a, Axl, TIM-1, and Tyro3 (Fig 1A) at levels consistent with human megakaryocytes *in vivo* [40–43]. Interestingly, we noted that approximately 15% of UT-7 cells express DC-SIGNR, a homologue of DC-SIGN. Expression of DC-SIGN but not DC-SIGNR has been observed in human megakaryocytes and platelets; however, this study uses reagents that do not distinguish between DC-SIGN and DC-SIGNR [43]. In our study, we used clones of antibodies that are specific to DC-SIGN or DC-SIGNR and do not cross-react with the other receptor (R&D Systems). To determine whether DENV enters UT-7 cells, UT-7 cells were infected with DENV-2 K0049 or DENV-2 16681 at various multiplicities of infection (MOI). Daily, post infection, UT-7 cells were collected, permeabilized, and stained for DENV envelope protein using the 4G2 monoclonal antibody. Dengue virus envelope protein was visualized in UT-7 cells infected with 16681 MOI 1, 16681 MOI 0.1, and K0049 MOI 1 with the peak occurring at day 1 post infection (Fig 1B). To eliminate the possibility that these results were reflective of the viral inoculum and not infected cells, we performed flow cytometry on UT-7 cells immediately post infection and cell washing (2 hours post infection). DENV envelope protein was not observed at this time point, indicating that the observed positive results are indeed due to DENV infection and not persistence of the initial inoculum. To corroborate our flow cytometry results, we performed immunofluorescence on infected UT-7 cells. Once again, we saw DENV envelope protein in UT-7 cells infected with 16681 MOI 1 and, to a lesser extent, in cells infected with K0049 MOI 1 (Fig 2). In both assays, UV inactivated K0049 was used as controls; UV inactivated 16681 was not used because UV inactivated K0049 represents a better negative control as K0049 is a more realistic viral strain for infection. No DENV envelope protein was detected in cells exposed to the UV inactivated K0049 control. Together these results indicate that DENV can enter and infect UT-7 cells.

**Fig 3:**
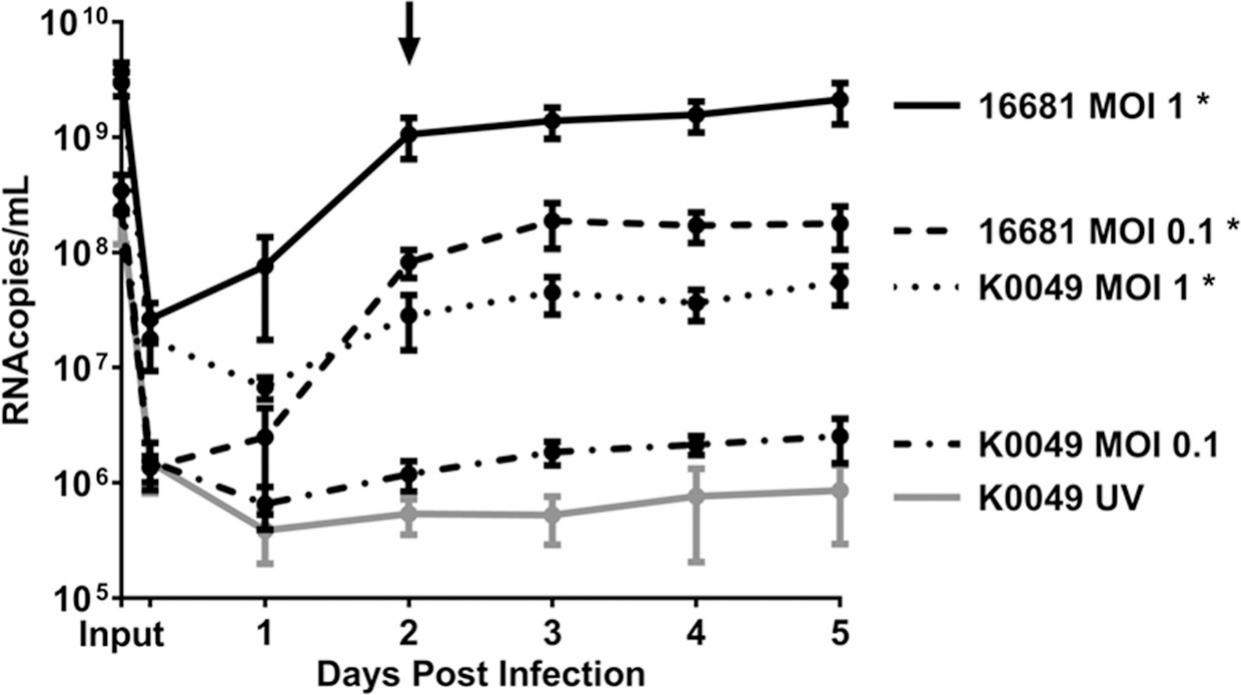
Dengue virus replicates in UT-7 cells. UT-7 cells were infected with DENV-2 16681 or DENV-2 K0049 at various MOIs. UT-7 cells incubated with UV-inactivated K0049 served as a control. Immediately following infection and daily post infection, supernatant samples were collected and DENV RNA was quantified via qRT-PCR. Data from three independent experiments are represented as the mean number of RNA copies per milliliter of cell supernatant. Error bars are 1 SEM. The arrow points to the timepoint at which we tested for and found infectious virus in all supernatants, except for those from cells treated with UV-inactivated K0049. Statistical significance was determined using a two-way ANOVA and multiple comparisons t-test (with Holm-Sidak correction). Statistical significance next to the virus strain name indicate that that infection was significantly different from the UV-inactivated control as assessed by two-way ANOVA. Significance at a specific timepoint indicates that the condition at that timepoint was significantly different from the UV-inactivated control via t-test but that the overall infection was not significantly different via ANOVA. * p<0.05; **p<0.01; *** p<0.005; ****p<0.001.

To determine whether DENV replicates in UT-7 cells, UT-7 cells were infected with DENV-2 16681 or DENV-2 K0049 at various MOIs. Each day post infection, cell supernatants were collected, and concentrations of viral RNA within these supernatants were determined via qRT-PCR. DENV RNA was present in the supernatants of UT-7 cells infected with 16681 MOI 1, 16681 MOI 0.1, and K0049 MOI 1 at a significantly higher amount than cells exposed to the K0049 UV inactivated control (Fig 3). Furthermore, the amount of DENV RNA present increased over time in the aforementioned conditions. Additionally, we observed persistence of viral RNA in the supernatants of UT-7 cells exposed to the K0049 UV inactivated control, and we were concerned that our results for all experimental groups may just reflect input virus. To distinguish between persistence of input virus and replication of viral genome, we “infected” cell-free tissue culture dishes in the same manner as the infection of UT-7 cells and assayed for viral RNA over time. Viral RNA concentrations from supernatants of UT-7 cells infected with 16681 MOI 1, 16681 MOI 0.1, K0049 MOI 1, and K0049 MOI 0.1 were significantly increased compared to levels found in cell-free wells (S1 Fig). Thus, we concluded that replication of the DENV genome occurred in UT-7 cells infected with 16681 MOI 1, 16681 MOI 0.1, and K0049 MOI 1.

The presence of DENV RNA in the supernatant does not indicate that infectious virus was present. To test whether infected UT-7 cells produced infectious virus, we performed endpoint dilution assays of UT-7 supernatants at peak infection (day 2 post infection) onto C6/36 cells. Following a 7 day incubation, infected C6/36 cells were visualized via immunofluorescence against the DENV envelope protein. Infectious virus was found in supernatants from UT-7 cells infected with either strain at either MOI, with the endpoint dilutions being 1:10,000 for 16681 MOI 1; 1:1,000 for 16681 MOI 0.1; 1:1,000 for K0049 MOI 1; and 1:100 for K0049 MOI 1. We were concerned that this infectious virus might have been inoculum that persisted in the media. Thus, we also looked for infectious virus immediately post infection and cell washing (2 hours post infection). Infectious virus was found at this timepoint with the endpoint dilutions being 1:1000 for 16681 MOI 1; 1:100 for 16681 MOI 0.1; 1:10 for K0049 MOI 1, and 1:10 for K0049 MOI 0.1. No infectious virus was found at either timepoint in supernatants from UT-7 cells exposed to K0049 UV inactivated virus. Infectious virus present in supernatants at 2 days post infection was significantly higher than infectious virus found in supernatants collected immediately post infection (2 hours post infection), indicating that the amount of DENV produced by infected UT-7 cells increased over time. These results indicate that DENV undergoes productive infection in UT-7 cells.

### Dengue virus infects primary human megakaryocytes

**Fig 4:**
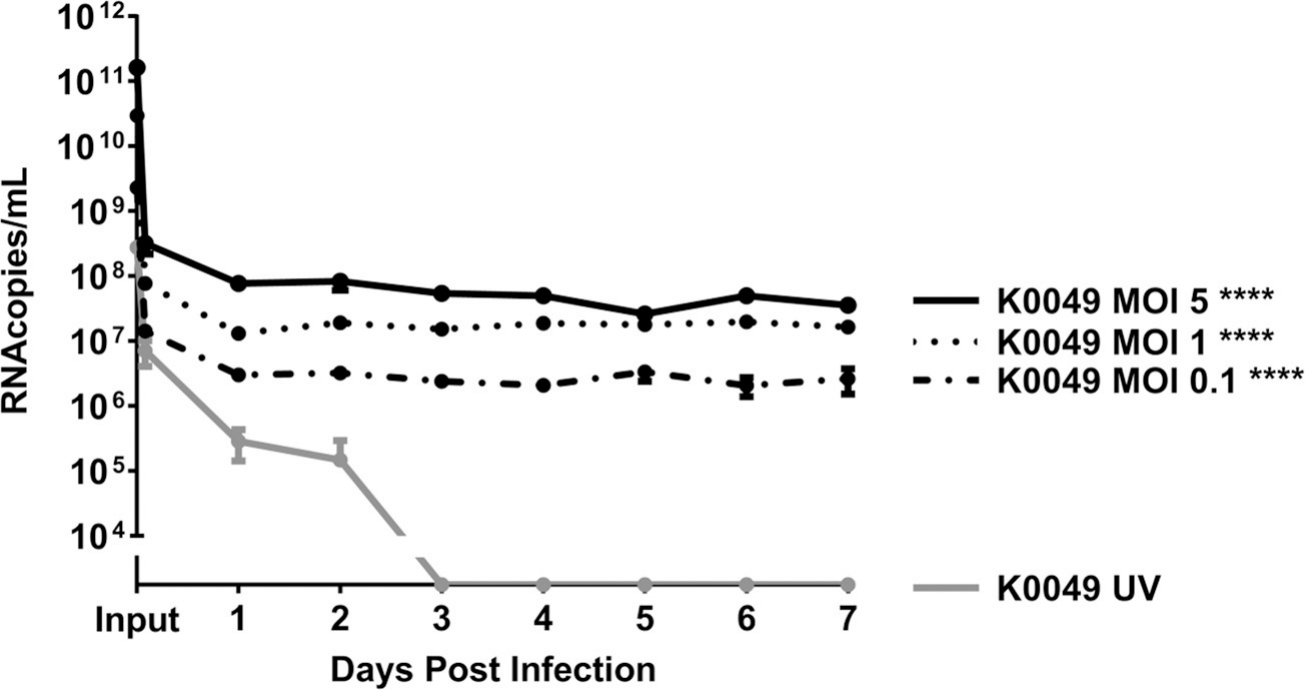
Dengue virus infects human megakaryocytes differentiated *ex vivo*. Human megakaryocytes were differentiated *ex vivo* from human CD34^+^ stem cells treated with TPO and other cytokines. Immature megakaryocytes (as determined by flow cytometry) were infected with DENV-2 K0049 at various MOIs. Following infection, cell supernatants were collected daily, and DENV RNA was quantified using qRT-PCR. Data from three independent experiments are represented as the mean number of RNA copies per milliliter of cell supernatant. Error bars are 1 SEM. Statistical significance was determined using a two-way ANOVA and multiple comparisons t-test (with Holm-Sidak correction). Statistical significance next to the virus strain name indicate that that infection was significantly different from the UV-inactivated control as assessed by two-way ANOVA. Significance at a specific timepoint indicates that the condition at that timepoint was significantly different from the UV-inactivated control via t-test but that the overall infection was not significantly different via ANOVA. * p<0.05; **p<0.01; *** p<0.005; ****p<0.001.

After determining that DENV infects and replicates in an *in vitro* model of human megakaryocytes (UT-7), we investigated whether DENV infects primary human megakaryocytes differentiated *ex vivo*. For this model, human CD34^+^ stem cells (obtained from human umbilical cord vein blood) were differentiated using a cytokine cocktail containing human thrombopoietin, SCF, IL-9, and IL-6 (Stem Cell Technologies) into immature megakaryocytes (determined by flow cytometry) [44]. These immature megakaryocytes were infected with DENV-2 K0049 at various MOIs, and DENV RNA was quantified in the cell supernatant via qRT-PCR. DENV-2 RNA was found in megakaryocyte supernatants up to 7 days post infection at significantly higher levels than megakaryocytes treated with UV-inactivated virus (Fig 4). The amount of viral RNA does not increase over time; however, viral RNA does persist in supernatants of infected cells while viral RNA concentration decreases over time in cells treated with UV-inactivated virus. This indicates that persistent infection and replication of DENV genome occurs in human megakaryocytes. We were unable to titrate for infectious virus as samples were limited due to cell longevity and size (a minimum of 200 μL sample is needed for infectious virus detection).

### Dengue virus infects human megakaryocytes in an *in vivo* model of infection

**Fig 5:**
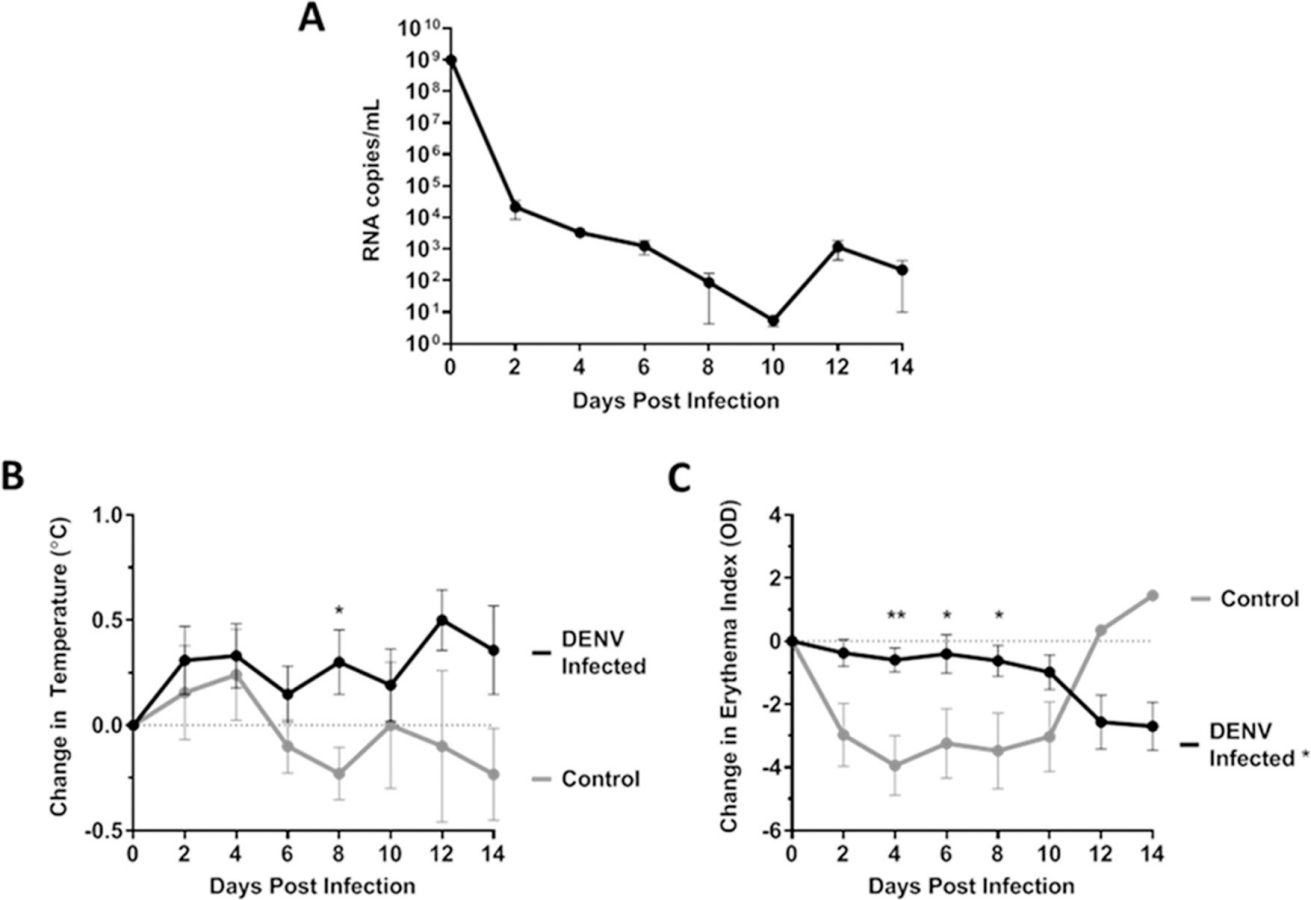
Hu-NSG mice have viremia and clinical signs of disease after DENV infection. (A-C) Hu-NSG mice were infected subcutaneously with 10^6^ PFU DENV/mouse. Viremia (n = 17 DENV infected) (A), temperature change (n = 25 DENV infected, n = 10 control) (B), and erythema change (n = 25 DENV infected, n = 10 control) (C) were measured throughout the infection. Data are represented as the mean number of RNA copies per milliliter of serum (A), mean change in temperature (°C) from baseline (B), or mean change in erythema index (O.D.) from baseline (C). Error bars are 1 SEM. (B-C) Statistical significance was determined using a two-way ANOVA and multiple comparisons t-test (with Holm-Sidak correction). Statistical significance next to the virus strain name indicate that that infection was significantly different from the UV-inactivated control as assessed by two-way ANOVA. Significance at a specific timepoint indicates that the condition at that timepoint was significantly different from the UV-inactivated control via t-test but that the overall infection was not significantly different via ANOVA. * p<0.05; **p<0.01; *** p<0.005; ****p<0.001.

After discovering that DENV infects human megakaryocytes in both *in vitro* and *ex vivo* models, we then used the hu-NSG mouse model, which possesses human immune cells, human megakaryocytes, and human platelets. Furthermore, hu-NSG mice exhibit clinical signs of dengue fever similar to those of humans [29, 45]. We infected hu-NSG mice via subcutaneous injection of DENV-1/VN/BiD-V1792 (n = 4), DENV-2 K0049 (n = 6), or DENV-3 7341/98 (n = 9). Preliminary studies using a DENV-4 strain at higher passage lever did not cause viremia or clinical signs. We were unable to obtain a low passage strain of DENV-4; therefore DENV-4 infected mice were not included in this study. Six saline-inoculated mice were used as controls. Sex, age, and engraftment levels of these mice are summarized in S1 Table. Mice were monitored every other day over the course of infection for viremia, fever, and erythema (Fig 5). DENV RNA was found in the blood of hu-NSG mice at all timepoints tested (Fig 5A). Infected mice consistently had a temperature above baseline and trended towards an increase over time, while the uninfected control mice showed the opposite (Fig 5B). Infected mice also maintained consistent erythema over the course of infection compared to baseline, while uninfected mice showed a significant decrease in erythema compared to infected mice (Fig 5C). We were unable to titrate infectious virus in serum because samples from the hu-NSG mice are too small for virus titration (a minimum of 200μL is need for infectious virus titration).Together, these results indicate that these mice were indeed infected by DENV and they showed clinical signs of infection.

**Fig 6:**
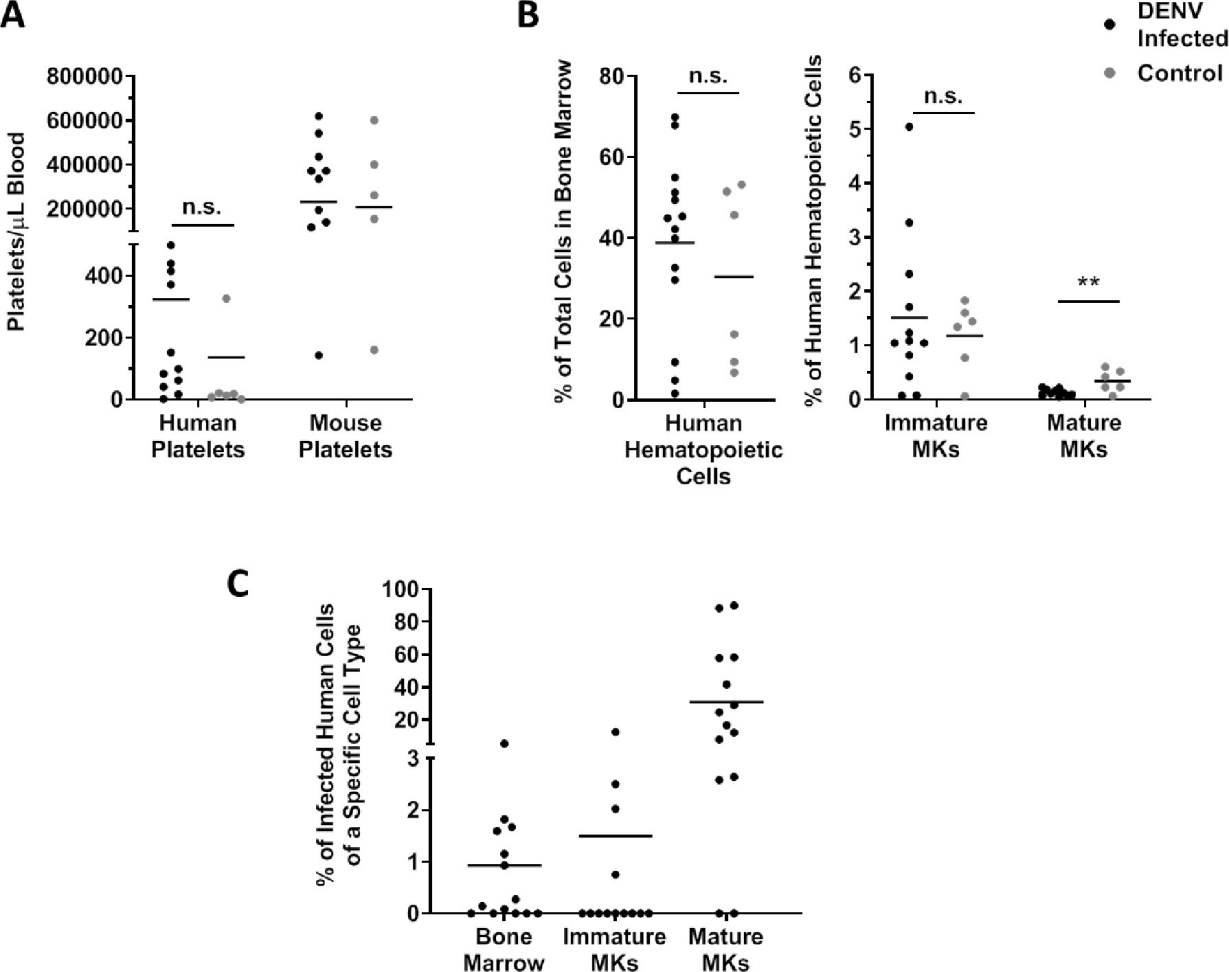
Human megakaryocytes are infected after DENV inoculation of Hu-NSG mice. (A) To assess platelet counts in Hu-NSG mice following DENV infection, blood was collected from mice at 10 days post infection, and platelets were counted via flow cytometry using antibodies specific for human and mouse platelets (n = 14 DENV infected, n = 6 control). The platelet count per milliliter of blood of each hu-NSG mouse are plotted. Horizontal lines depict the group mean. Outliers were removed via ROUT analysis, and statistical significance was assessed using a t-test. (B) To determine whether bone marrow and megakaryocyte suppression occurred in infected hu-NSG mice, bone marrow was collected from mice upon euthanasia. Cell type was determined via flow cytometry (n = 14 DENV infected, n = 6 control). Data are represented as the percentage of either total bone marrow cells or human hematopoietic cells. Values from individual hu-NSG mice are plotted with horizontal lines depicting the group mean. Outliers were removed via ROUT analysis, and statistical significance was assessed using a t-test. (C) To determine whether infected megakaryocytes were in the bone marrow of hu-NSG mice, bone marrow was collected from mice upon euthanasia. Cell type and infectious status were determined via flow cytometry (n = 14 DENV infected, n = 6 control). Data are represented as the percentage of infected cells of a given cell type. Values from individual hu-NSG mice are plotted with horizontal lines depicting the group mean. * p<0.05; **p<0.01; *** p<0.005; ****p<0.001 MK, Megakaryocyte.

We next investigated whether thrombocytopenia occurred in infected hu-NSG mice by using flow cytometry to count both human and mouse platelets at 10 days post infection, which is when peak thrombocytopenia occurs based on our previous studies using this model [29, 46]. There were no significant differences in human or mouse platelet counts between infected or control mice (Fig 6A). In past studies by our lab and others, thrombocytopenia does occur in DENV-2-infected hu-NSG mice [24, 29, 45, 46]. The clinical signs we observed in these mice did appear to be milder than those of our previous studies, using dengue serotype 2 viruses alone, and here we included serotype 1 and 3 infections, which seemed to produce less severe infections. Additionally, the amount of human platelets produced in the hu-NSG mouse model varies by individual animal, and this variation appears to be related to the number and efficacy of mouse derived macrophages that persist in the hu-NSG mice following the engraftment procedure [47, 48]. We were unaware of this before beginning this study and did not take human platelet count into consideration when creating experimental groups of hu-NSG mice. Despite the lack of significant differences in platelet counts, the frequency of human mature megakaryocytes in the bone marrow was significantly decreased in DENV-infected mice. However, the frequencies of total human cells and human immature megakaryocytes were not significantly different between infected and control mice. Sridharan *et al* also reported a significant decrease in human megakaryocytes in the bone marrow of infected hu-NSG mice but did not distinguish between immature and mature megakaryocytes nor comment on changes in the human cell population in the bone marrow [24]. Finally, we used flow cytometry to determine whether human bone marrow cells and megakaryocytes were targets of DENV during hu-NSG infection. We discovered that 1% of total bone marrow cells, 1.5% of human immature megakaryocytes, and 35% of human mature megakaryocytes were infected with DENV (Fig 6B). Together, these data indicate that DENV does indeed reach the bone marrow and infects mainly mature megakaryocytes during the course of disease.

### Dengue virus does not affect megakaryocyte viability *in vitro*

**Fig 7:**
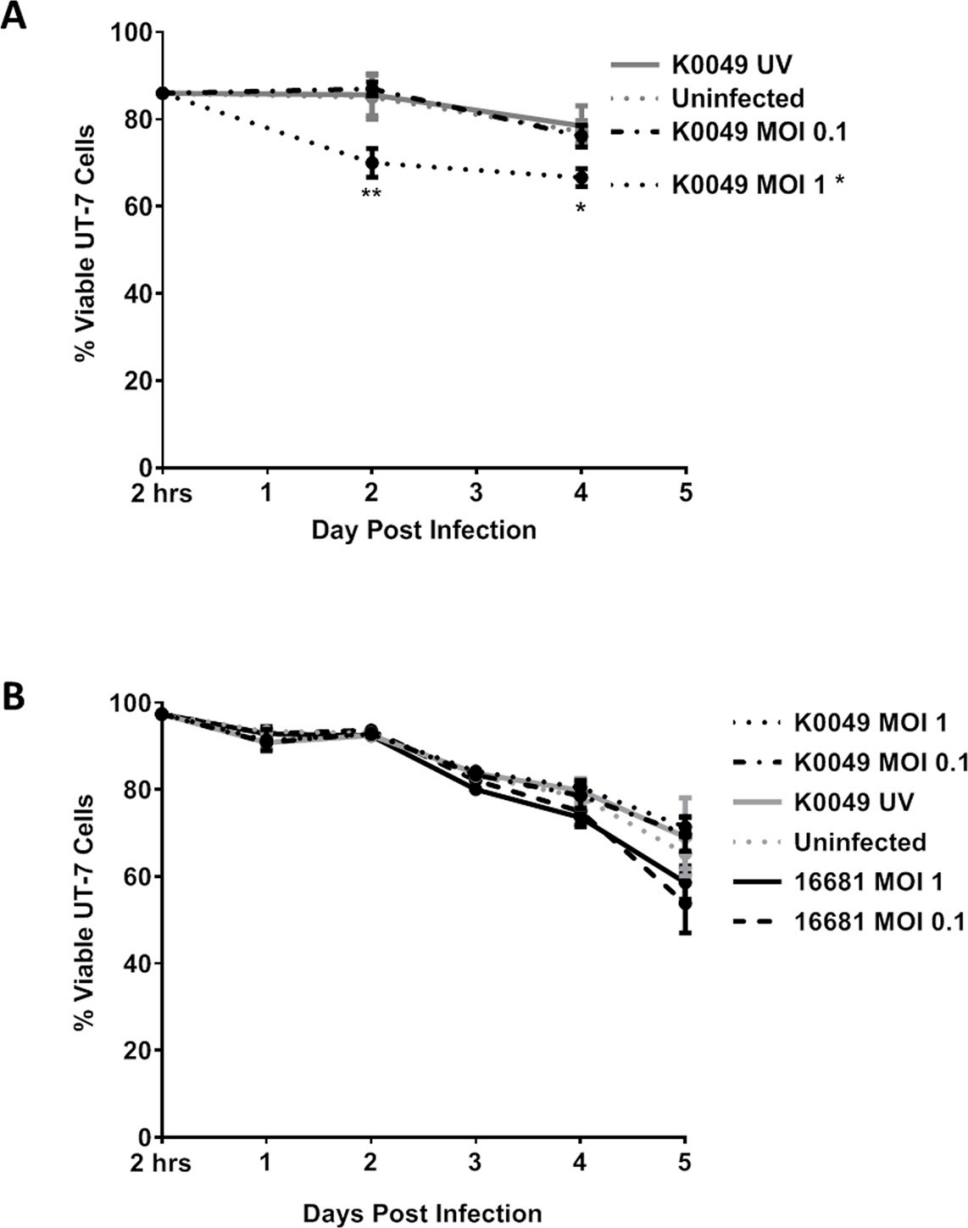
Dengue virus infection does not affect UT-7 cell viability. Viability of UT-7 cells infected with DENV was assessed every other day via trypan blue (A) or daily via flow cytometry (B). Data from three independent experiments are represented as the mean percentage of viable UT-7 cells. Error bars are 1 SEM. Statistical significance was determined using a two-way ANOVA and multiple comparisons t-test (with Holm-Sidak correction). Statistical significance next to the virus strain name indicates that that infection was significantly different from the uninfected control as assessed by two-way ANOVA. Significance at a specific timepoint indicates that the condition at that timepoint was significantly different from the uninfected control via t-test. * p<0.05; **p<0.01; *** p<0.005; ****p<0.001.

Since DENV infects human megakaryocytes *in vitro*, *ex vivo*, and *in vivo* models of infection and DENV decreases mature megakaryocyte populations *in vivo*, we investigated the mechanism of DENV depletion of megakaryocytes. Previous studies showed that exposure to DENV *in vivo* and *in vitro* results in reduced viability of megakaryocytes; however, they did not investigate whether reduced viability was due to the direct DENV infection of megakaryocytes or some other mechanism [10, 24, 49]. Thus, we assessed whether DENV infection of megakaryocytes *in vitro* reduces viability. We first assessed viability of UT-7 cells following K0049 infection via trypan blue exclusion (Fig 7A). Uninfected UT-7 cells served as a negative control. Viability of UT-7 cells infected with K0049 MOI 1 was significantly reduced compared to uninfected controls at 2 and 4 days post infection. We were concerned that trypan blue exclusion may be more susceptible to human error and bias than other viability assays, so we assessed UT-7 cell viability via flow cytometry. UT-7 cells were infected with K0049 or 16681 at various MOIs. Daily, cells were collected and viability was assessed (Fig 7B). Overall, viability of infected cells, as measured by flow cytometry, did not differ significantly compared to the uninfected controls, with the exception of UT-7 cells infected with 16681 MOI 0.1 on day 5 post infection. Cells in all conditions experienced a significant change in viability over time, most like caused by unfavorable culture conditions post infection. During growth, UT-7 cells were split and media changed every 3 days, whereas during infection cells were not split and only 10% of the media were changed daily. These changes were made to prevent removing large amounts of media containing virus post infection but may have inadvertently affected viability. These results are very important because they indicate that DENV alone is not responsible for megakaryocyte depletion and that some other mechanism, perhaps host-mediated, is involved.

## Discussion

In this study, we sought to investigate a mechanism for DENV-induced megakaryocyte suppression by addressing whether DENV directly infects human megakaryocytes. We determined that DENV indeed infects human megakaryocytes *in vitro*, *ex vivo*, and *in vivo* models of infection. We observed that DENV significantly decreases populations of mature megakaryocytes *in vivo*, in humanized mice bone marrow. We also observed that DENV does not affect cell viability *in vitro*, indicating that the depletion of megakaryocytes *in vivo* is likely due to a mechanism other than direct induction of cell death. To our knowledge, this is the first report of DENV infection of human megakaryocytes in an *in vivo* model. While other studies have observed DENV infected cells in the bone marrow and investigated the effects of DENV infection on the viability of other human megakaryocyte-like cells, none of them investigated whether DENV infects human megakaryocytes in an animal model. A recent study showed DENV infection of human megakaryocytes that were differentiated *ex vivo;* however, infection was only assessed at 18 hours post infection, with DENV-2 strain 16681 and focused on measuring upregulation of interferon stimulated genes [50]. In contrast, our study characterized DENV infection in human megakaryocytes in 3 different model systems, and brings novel findings and conclusions to the field of DENV pathogenesis research.

Sridharan *et al* are the only group to have previously measured human megakaryocytes and platelets in the hu-NSG model of DENV infection [24]. In their study, hu-NSG mice experienced a significant decrease in human, but not mouse, platelets and human megakaryocytes following infection, which is consistent with other reports of DENV infection in various humanized mouse models [51–56]. In our study, neither human nor mouse platelets were significantly decreased in DENV-infected Hu-NSG; however, we used different strains of DENV, low passage clinical isolates, compared to Sridharan *et al*, who used New Guinea C. New Guinea C, is a highly-passaged, lab-adapted strain that differs structurally from wild-type viruses, including a heat stability phenotype. [57]. Repeated passaging modifies viral virulence, pathogenesis, and tropism, thus rendering these strains clinically irrelevant [58, 59]. Furthermore, we used DENV strains from three different serotypes (DENV-1,2,3); these serotypes differ in their propensity to cause severe disease in humans [60–62], possibly explaining why we did not detect significant thrombocytopenia in some of our mice.

In our study, we observed a decrease in mature megakaryocytes in the bone marrow following DENV infection. Sridharan *et al* concluded that DENV infection reduces megakaryocyte populations, but did not distinguish between immature and mature megakaryocytes [24]. This distinction is important because immature and mature megakaryocytes are functionally different; immature megakaryocytes produce large amounts of RNA and proteins for platelet production and begin platelet assembly, while mature megakaryocytes finish platelet assembly and release platelets into the blood [9]. Furthermore, the number of immature and mature megakaryocytes in the bone marrow may differ over the course of human infection. Bierman and Nelson reported that bone marrow from patients with severe DENV infection had little to no megakaryocytes upon hospitalization [10], and during convalescence, populations of immature megakaryocytes rebounded before mature megakaryocytes [10]. The kinetics of megakaryocyte depletion upon DENV infection, specifically in regards to immature and mature megakaryocytes, are currently unknown.

In this study, we determined that DENV infection decreases populations of mature megakaryocyte in the bone marrow of hu-NSG mice, which is consistent with other studies in humans, rhesus macaques and mice [10, 24, 26]. Additional studies have shown that exposure to DENV reduces viability and proliferation of megakaryocytes in cell culture [25, 49]. However, when we infected human megakaryocytes *in vitro* (UT-7 cells), we did not notice a significant change in viability compared to uninfected controls, with the exception of DENV-2 16681 at MOI 0.1 (Fig 7). Basu *et al* and Clark *et al* reported reduced viability and proliferation following DENV infection of human megakaryocytes differentiated *ex vivo* and unfractionated bone marrow samples obtained from healthy humans and rhesus macaques, respectively [25, 49]. The virus strains used were either cultured in mouse brains or highly passaged, which are likely to be pathogenic in ways that are not reflective of natural infection. Even though we did not observe reduced viability of DENV infected megakaryocytes *in vitro*, this does not preclude the possibility that DENV infection indirectly reduces megakaryocyte viability, perhaps through immune responses. For example, megakaryocyte-like cells infected with Hantaan virus upregulated MHC class I to the cell surface, potentially allowing for increased killing of infected megakaryocytes by cytotoxic T lymphocytes [63]. Additionally, IFNγ, which is found at significantly high levels in the serum of DENV infected individuals [64–66], has been associated with exhaustion of hematopoietic stem cells and bone marrow suppression [67]. Immune reactions like these may have occurred in the aforementioned *in vivo* DENV infections and to a certain extent in *ex vivo* DENV infections of bone marrow samples, whereas they would not have occurred in our *in vitro* infection of human megakaryocytes.

In addition to DENV, other hemorrhagic fever viruses such as Hantaan virus and Junín virus (the causative agents of hemorrhagic fever with renal syndrome and Argentine hemorrhagic fever, respectively) infect megakaryocytes. In humans, Hantaan virus antigen has been found in the bone marrow of individuals suffering from hemorrhagic fever with renal syndrome, with approximately 20% of their megakaryocytes expressing this viral antigen [68]. Further experiments revealed that Hantaan virus infected primary human megakaryocytes and resulted in the upregulation of MHC Class I on infected megakaryocytes, promoting megakaryocyte death by cytotoxic T lymphocytes [63]. In human cases of Argentine hemorrhagic fever, infected megakaryocytes in the bone marrow have not yet been reported; however, in a guinea pig model of Junín virus infection, infected megakaryocytes were visualized by immunohistochemistry and electron microscopy [69, 70]. Further, human primary megakaryocytes have successfully been infected with Junín virus [71].

Infection of megakaryocytes may be beneficial to DENV and other hemorrhagic fever virus replication. Megakaryocytes modulate hematopoietic stem cell quiescence, regeneration, and proliferation through the production of various cytokines [19–21]. Additionally, megakaryocytes maintain plasma cell (terminally differentiated, antibody-secreting B cells) niches in the bone marrow [22, 23]. If infection suppresses or alters megakaryocyte function, then differentiation of immune cells and production of antibodies needed to clear the infection may be affected. Lastly, platelets, themselves, can act as immune mediators. Platelets can recruit white blood cells, bind and present pathogens to antigen presenting cells, and produce pro-inflammatory, anti-inflammatory, or activating cytokines [72–75]. If infection of megakaryocytes reduces platelet counts or causes platelet dysfunction, then the immune system functions of platelets may be diminished. Thus, infection of megakaryocytes may suppress the immune system, ultimately leading to increased viral replication or pathogenesis.

Dengue virus is a significant global health burden that will increase in scope as climate change and international travel continue, yet we do not have a complete understanding of the mechanisms behind DENV pathogenesis. In this study, we showed that DENV infects human megakaryocytes *in vitro*, *ex vivo*, and *in vivo*, potentially explaining how megakaryocyte suppression occurs during DENV infection. These results will help inform the development of specific DENV therapies, which may also be useful for other viral hemorrhagic fevers that target megakaryocytes. The results presented here serve as a starting point for future studies into the mechanisms and consequence of DENV-induced bone marrow suppression.

## Supporting information

S1 Table Hu-NSG mice used in DENV infection of megakaryocytes study. This table describes the sex, age, and engraftment levels of hu-NSG mice used in this study. Hu-NSG mice are sorted by experimental group.
(DOCX)

S1 Text Gating strategies for flow cytometry experiments. This appendix lists the gating strategies for all flow cytometry experiments within this dissertation, with the exception of the putative DENV receptor flow cytometry in UT-7 cells. (In the receptor flow cytometry assay, cells were singly stained for receptors; thus, no gating strategy was needed). Gating strategies are presented as leveled lists. Each level represents a gated population of cells. Indented populations are contained entirely within the previous population of a higher order. For example, in the gating strategy for UT-7 infection and viability, the SSC singlet population is comprised only of cells within the FSC singlet population. The populations 4G2+ and 4G2- are comprised only of cells that were ghost-dye negative. Levels that contain two markers (e.g. CD41a+CD42b+) were gated via quadrant gate, while the others were gated via histogram or single gates on a two-dimensional plot. All cell surface markers mentioned are human unless otherwise designated (m = mouse; h = human).
(DOCX)

S1 Fig Persistence of DENV RNA in cell free media. An infection identical to UT-7 cell infection, with the exception that there were no cells, was set up. Samples from these cell-free infections were collected daily, and DENV RNA was assessed via qRT-PCR. These data are compared to data from UT-7 cell infections. Data from three independent experiments are represented as the mean number of RNA copies per milliliter of cell supernatant. Error bars are 1 SEM. Statistical significance was determined using a two-way ANOVA, and statistical significance is marked next to the virus strain.
(TIF)
