# Supplementary material for: Dengue viruses infect human megakaryocytes, with probable clinical consequences
Source: PLoS Negl Trop Dis. 2019 Nov 25;13(11):e0007837. doi: 10.1371/journal.pntd.0007837 (PMC6901235; doi:10.1371/journal.pntd.0007837)
Supplement: S1 Table — This table describes the sex, age, and engraftment levels of hu-NSG mice used in this study. Hu-NSG mice are sorted by experimental group. (DOCX) [file pntd.0007837.s001.docx]

| **Virus** | **Mouse ID** | **Sex** | **Age (weeks)** | **Engraftment (%)** |
| --- | --- | --- | --- | --- |
| DENV-1 | 729 | F | 14 | 42.7 |
| DENV-1 | 730 | F | 14 | 16.2 |
| DENV-1 | 731 | F | 14 | 14.5 |
| DENV-1 | 732 | F | 14 | 17.9 |
|  |  |  |  |  |
| DENV-2 | 719 | F | 6 | 60 |
| DENV-2 | 720 | F | 6 | 47 |
| DENV-2 | 724 | M | 6 | 77 |
| DENV-2 | 725 | M | 6 | 32 |
| DENV-2 | 727 | M | 6 | 72 |
| DENV-2 | 728 | M | 6 | 61 |
|  |  |  |  |  |
| DENV-3 | 550 | M | 33 | 74 |
| DENV-3 | 618 | F | 21 | 59 |
| DENV-3 | 654 | F | 13 | 43 |
| DENV-3 | 655 | F | 13 | 24 |
| DENV-3 | 791 | F | 11 | 38.6 |
| DENV-3 | 792 | M | 11 | 35 |
| DENV-3 | 793 | M | 11 | 28 |
| DENV-3 | 795 | M | 11 | 27 |
| DENV-3 | 796 | M | 11 | 26 |
|  |  |  |  |  |
| Control | 721 | F | 6 | 90.5 |
| Control | 733 | F | 14 | 13.4 |
| Control | 745 | F | 14 | 17.6 |
| Control | 776 | M | 12 | 14 |
| Control | 785 | M | 12 | 10.3 |
| Control | 786 | M | 12 | 15.4 |
