# Supplementary material for: Dengue viruses infect human megakaryocytes, with probable clinical consequences
Source: PLoS Negl Trop Dis. 2019 Nov 25;13(11):e0007837. doi: 10.1371/journal.pntd.0007837 (PMC6901235; doi:10.1371/journal.pntd.0007837)
Supplement: S1 Text — This appendix lists the gating strategies for all flow cytometry experiments within this dissertation, with the exception of the putative DENV receptor flow cytometry in UT-7 cells. (In the receptor flow cytometry assay, cells were singly stained for receptors; thus, no gating strategy was needed). Gating strategies are presented as leveled lists. Each level represents a gated population of cells. Indented populations are contained entirely within the previous population of a higher order. For example, in the gating strategy for UT-7 infection and viability, the SSC singlet population is comprised only of cells within the FSC singlet population. The populations 4G2+ and 4G2- are comprised only of cells that were ghost-dye negative. Levels that contain two markers (e.g. CD41a+CD42b+) were gated via quadrant gate, while the others were gated via histogram or single gates on a two-dimensional plot. All cell surface markers mentioned are human unless otherwise designated (m = mouse; h = human). (DOCX) [file pntd.0007837.s002.docx]

**UT-7 Infection and Viability Status (**Figure 1, Figure 7**)**

1. Forward Scatter (FSC) Singlets
   1. Side Scatter (SSC) Singlets
      1. Size Exclusion
         1. Ghostdye-
            1. 4G2+ Live, Infected Cells
            2. 4G2- Live, Uninfected Cells
         2. Ghostdye+ Dead Cells

**Megakaryocyte Differentiation** (preparation for infection in Figure 4)

1. FSC Singlets
   1. SSC Singlets
      1. Size Exclusion
         1. CD41a+CD42b+ Mature Megakaryocytes
         2. CD41a+CD42b- Immature Megakaryocytes
         3. CD41a-CD42b+
         4. CD41a-CD42b-

**Infected Megakaryocytes in Hu-NSG Mice (**Figure 6**)**

1. FSC Singlets
   1. SSC Singlets
      1. Size Exclusion
         1. CD45+ Bone Marrow Cells
            1. 4G2+ DENV-Infected Bone Marrow Cells
            2. 4G2-
            3. CD41a+CD42b+ Mature Megakaryocytes

4G2+ DENV-infected Mature Megakaryocytes

4G2-

- - - - 1. CD41a+CD42b- Immature Megakaryocytes

4G2+ DENV-Infected Immature Megakaryocytes

4G2-

- - - - 1. CD41a-CD42b+

4G2+

4G2-

- - - - 1. CD41a-CD42b-

4G2+

4G2-

**Platelet Counts in Hu-NSG Mice (**Figure 5**)**

1. FSC Singlets
   1. SSC Singlets
      1. CountBright Beads Counting Beads
      2. Size Exclusion
         1. mCD41+hCD41+
         2. mCD41+hCD41- Mouse Platelets
         3. mCD41-hCD41+ Human Platelets
         4. mCD41-hCD41-
