# Supplementary figures and images for: Dengue viruses infect human megakaryocytes, with probable clinical consequences
Source: PLoS Negl Trop Dis. 2019 Nov 25;13(11):e0007837. doi: 10.1371/journal.pntd.0007837 (PMC6901235; doi:10.1371/journal.pntd.0007837)

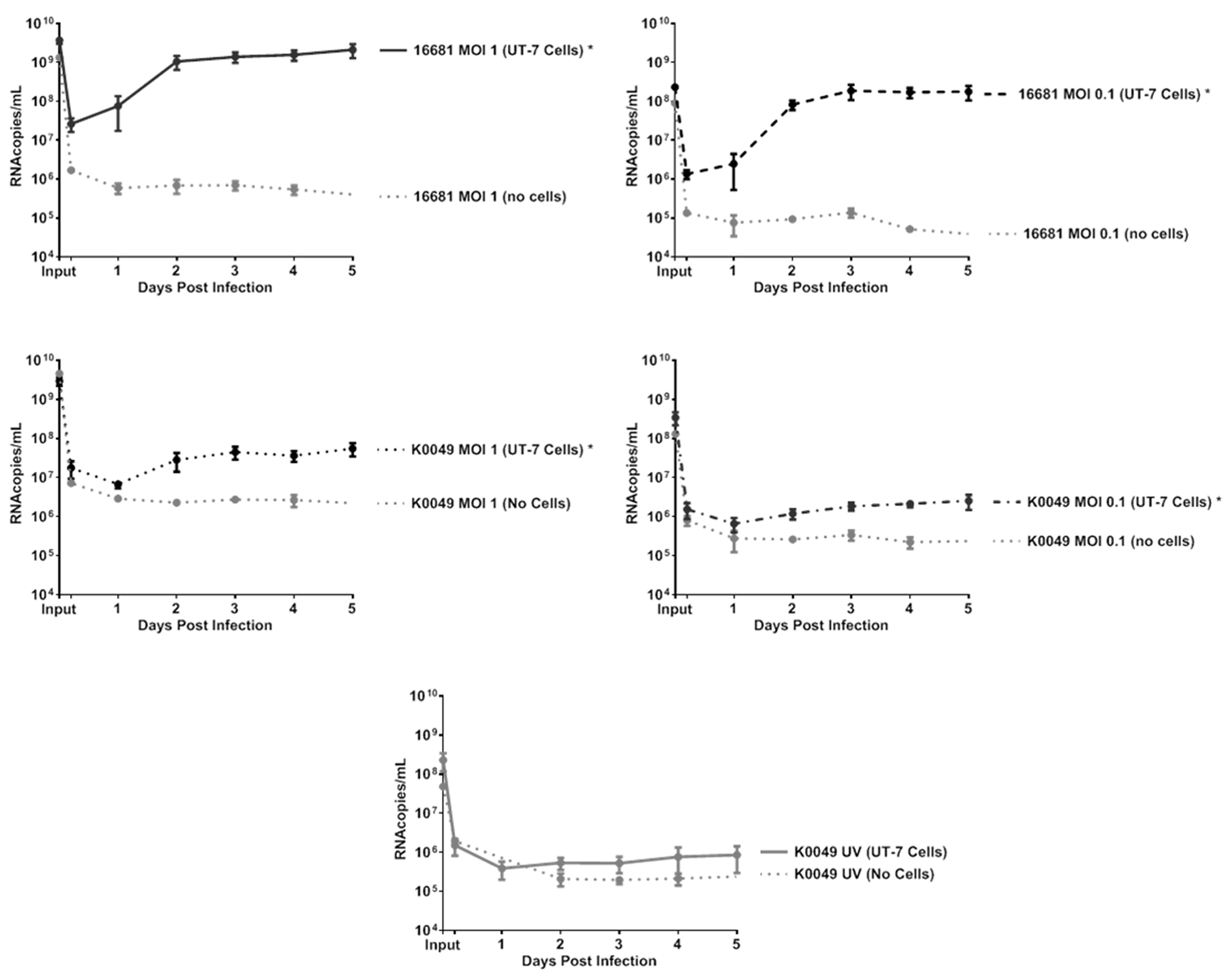

Supplement: S1 Fig — An infection identical to UT-7 cell infection, with the exception that there were no cells, was set up. Samples from these cell-free infections were collected daily, and DENV RNA was assessed via qRT-PCR. These data are compared to data from UT-7 cell infections. Data from three independent experiments are represented as the mean number of RNA copies per milliliter of cell supernatant. Error bars are 1 SEM. Statistical significance was determined using a two-way ANOVA, and statistical significance is marked next to the virus strain. (TIF) [file pntd.0007837.s003.tif]
